# Point-of-Care Ultrasound to Evaluate Gastric Volumes During Gastric and Post-Pyloric Enteral Feeds in Infants and Children Undergoing Mechanical Ventilation: A Pilot Study to Assess Feasibility Study

**DOI:** 10.14740/jocmr6506

**Published:** 2026-03-26

**Authors:** Timothy Montet, Ada Lin, Sibelle Aurelie Yemele Kitio, Joseph D. Tobias, Alok Moharir

**Affiliations:** a Department of Anesthesiology & Pain Medicine, Nationwide Children’s Hospital, Columbus, OH, USA; b Division of Pediatric Critical Care Medicine, Nationwide Children’s Hospital and Department of Pediatrics, The Ohio State University College of Medicine, Columbus, OH, USA; c Department of Anesthesiology & Pain Medicine, The Ohio State University College of Medicine, Columbus, OH, USA

**Keywords:** Point-of-care ultrasound, Enteral feedings, Aspiration, Ultrasonography

## Abstract

**Background:**

In all acutely ill patients, adequate nutrition is essential to restore physiologic homeostasis and improve outcomes. Whenever feasible, the enteral route is preferred. In various settings, post-pyloric (nasoduodenal (ND) or nasojejunal (NJ)) feeds may be preferred not only to accelerate attainment of goal feeding volumes, but also to limit gastric volumes in order to decrease the potential risk of aspiration. The current study uses point-of-care ultrasound (POCUS) to evaluate gastric volume and content during enteral feedings in pediatric-aged patients receiving mechanical ventilation in the pediatric intensive care unit (ICU).

**Methods:**

Gastric POCUS was performed to evaluate gastric contents in pediatric ICU patients, aged 0–18 years, receiving either gastric or post-pyloric enteral feedings at ≥ 50% goal. The patients were endotracheally intubated and receiving mechanical ventilation.

**Results:**

The study cohort included 45 patients, 29 receiving nasogastric (NG) feeds and 16 receiving NJ feeds. The majority of patients (81%) receiving post-pyloric feeds had gastric volumes ≤ 0.4 mL/kg and none had volumes ≥ 2 mL/kg while more than half of NG-fed patients (53%) had gastric volumes ≥ 2 mL/kg. Only three of 18 patients (18.8%) receiving NJ feeds had a gastric volume greater than 0.4 mL/kg. When grading the aspiration risk, there was a higher aspiration risk with NG feeds compared to NJ feeds (33/39 versus 0/18, P < 0.001).

**Conclusion:**

Gastric volumes and hence the potential aspiration risk is decreased in patients receiving post-pyloric compared to NG feeds.

## Introduction

Enteral nutrition support is essential in both medical and surgical patients cared for in pediatric and adult intensive care units (ICUs) [1–5]. Upon admission to an ICU, up to 30% of patients are considered malnourished and that percentage can increase to 60% during an ICU stay due to increased metabolic demands and decreased delivery of nutrition. Enteral nutrition is routinely recommended over parenteral nutrition whenever feasible as enteral nutrition maintains gut integrity, improves immune function, decreases infection risk, and is associated with an overall lower cost. When enteral nutrition is chosen, either nasogastric (NG) or post-pyloric (nasoduodenal (ND) or nasojejunal (NJ)) routes for enteral nutrition can be used.

Post-pyloric placement of a feeding tube is thought to allow for enteral feeds to be started in patients where aspiration risk is high (e.g., patients in respiratory failure on non-invasive ventilation) or in patients with neurocognitive delays and subsequent aspiration risks due to impaired gastric emptying. Despite this, our clinical experience suggests that many ICU patients may have NJ feedings interrupted or withheld for procedures for the same amount of time as those that are receiving NG feeds as there are limited clinical data evaluating gastric volumes during NJ feeding regimens.

Point-of-care ultrasonography (POCUS) has been recognized as a useful tool to evaluate the gastric contents and volume in various clinical settings [6–9]. In the perioperative setting, gastric POCUS and the evaluation of gastric contents may identify which patients are at risk for aspiration and therefore guide anesthetic induction or airway management technique such as a rapid sequence intubation (RSI). Additionally, proof that the gastric volumes are minimal using POCUS may allow continuation of post-pyloric feeds during procedures or planned liberation from mechanical ventilation, thereby limiting *nil per os* (NPO) times and improving total nutritional intake.

Despite the theoretical assumption that gastric volumes may be less with post-pyloric versus NG feeds, there are no previous studies using POCUS to assess gastric volumes during enteral feeding in infants and children in the ICU setting. The current study uses gastric POCUS to compare gastric volume/contents and hence the potential for aspiration in pediatric-aged patients receiving either NG or post-pyloric feeds during mechanical ventilation in the pediatric ICU. Our hypothesis was that gastric residual volumes would be minimal in patients receiving post-pyloric enteral feeds.

## Materials and Methods

This prospective study was approved by the Institutional Review Board (IRB) of Nationwide Children’s Hospital (STUDY00001377) and registered at clinicaltrials.gov (NCT04612348). This study was conducted in compliance with the ethical standards of our IRB on human subjects as well as with the Helsinki Declaration.

The study population included pediatric ICU patients, aged 0–18 years, who were endotracheally intubated and receiving mechanical ventilation for less than 14 days. The patients were receiving feeds at ≥ 50% goal. Following informed written consent from a parent or guardian, gastric ultrasound was performed on the eligible patient. Data collected included demographic data (age, gender, weight, height), type of feeding (NG or post-pyloric or NJ), rate of feeding (calculated as percent of goal volume), pediatric risk of mortality (PRISM) score, and comorbid conditions. Exclusion criteria included patients that could not be moved, those with significant gastrointestinal conditions affecting motility, and patients intubated for more than 14 days.

**Figure 1:**
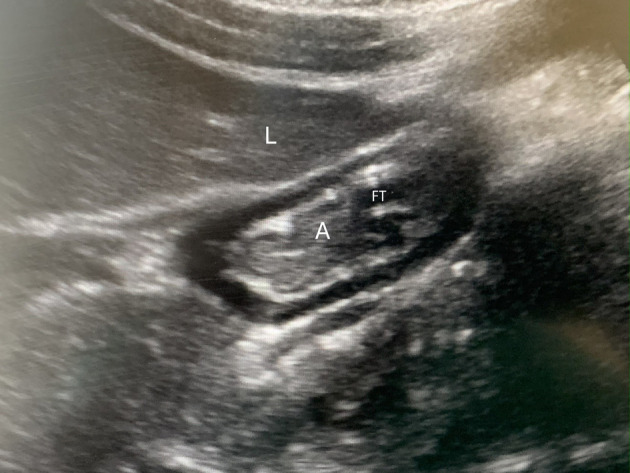
Gastric ultrasound in a patient receiving post-pyloric feedings via feeing tube (FT) showing the collapsed antrum (A) with the classic “bull’s eye appearance,” positioned under the liver (L).

Proper placement of the feeding tube (NG or NJ) was confirmed on the morning radiograph. Ultrasound studies were obtained up to three times on each patient on different days, based on availability of the authors trained in POCUS. Gastric ultrasound was performed in the pediatric ICU by an investigator proficient in diagnostic POCUS. A Sonosite X-Porte (Sonosite Inc. USA) machine with a low frequency (3–8 MHz) curvilinear probe was utilized. The ultrasound was performed with the child lying in the supine position followed by the right lateral decubitus (RLD) position. In situations where it was not feasible to place the patient in the right lateral decubitus position, the patient was placed in a 45° semi-recumbent position. The gastric antrum was identified in a sagittal plane between the left lobe of the liver and the pancreas at the level of the descending aorta and superior mesenteric artery (Fig. 1) [10]. Qualitative and quantitative measurements of the gastric antrum were recorded. The quantitative exam was performed using the cross-sectional area of the gastric antrum. The cross-sectional area (CSA) of the antrum was measured with a free-tracing method to follow the outer margin of the antrum corresponding to the serosal layer. The gastric volume was calculated using the following formula: Gastric volume (mL/kg) = [−7.8 + 0.035 × CSA (mm^2^) + 0.127 × age (months)]/body weight (kg)]. The qualitative and quantitative measurements were performed using a three-point grading scale as described by Spencer et al [6]. Grade 0 includes no fluid visible in the gastric antrum; grade 1 includes clear fluid visualized with volume less than 1.5 mL/kg; and grade 2 includes clear fluid visualized with a volume ≥ 1.5 mL/kg. A high risk of aspiration was defined as grade 2 or the presence of any solid or thick liquid on exam.

### Data presentation and statistical analysis

Continuous data were presented as a mean ± standard deviation or median (interquartile range). Categorical variables were presented as frequencies and percentages. Age and weight differences between the groups were compared using Wilcoxon rank-sum test while gender differences were compared using a Fisher’s exact test. Gastric volumes between the two groups were also compared with a Wilcoxon rank-sum test while the distributions of gastric volumes among the three volume categories (mL/kg) were compared with a Fisher’s exact test. All statistical analyses were performed using Stata version 18 (StataCorp, College Station, TX). Statistical significance was determined to be a P value < 0.05.

## Results

The study cohort included 45 patients, 29 receiving NG feeds and 16 receiving NJ feeds. The demographic data and patient characteristics are listed in Table 1. No significant difference was noted when comparing the NG and NJ groups. A total of 39 scans were performed in the 29 patients receiving NG feeds and 18 scans were performed in the 16 patients receiving NJ feeds. The percentage of patients receiving ≥ 50% of goal feeds at the time of the ultrasound was 89.2% in the NG group and 77.8% in the NJ groups.

**Table 1:** Demographic Characteristics of the Study Population

Characteristics	Nasogastric	Post-pyloric (NJ or ND)	Entire cohort
Study population (number and %)	29 (64.4)	16 (35.6)	45 (100)
Age (months)*	4 (2, 16)	12 (4, 122)	6 (2, 48)
Weight (kg)**	7.6 (5.5, 12.9)	10.1 (5.0, 37.3)	7.9 (5.0, 15.2)
Height (cm)	60 (53, 77)	76.1 (57.5, 129.5)	62 (54, 79)
Gender^+^			
Female	7 (24.1)	6 (37.5)	13 (28.9)
Male	22 (75.9)	10 (62.5)	32 (71.1)
PRISM category			
Low risk	21 (72.4)	8 (50.0)	29 (64.4)
Moderate risk	5 (17.2)	6 (37.5)	11 (24.4)
Unknown	3 (10.3)	2 (12.5)	5 (11.1)

Data are presented as the frequency (percentages) and median (interquartile ranges). PRISM category definition: ≤ 10 = low risk; 11 to ≤ 20 = moderate risk; >20 = high risk. *P = 0.155; **P = 0.420; ^+^P = 0.494 when comparing nasogastric to post-pyloric groups. ND: nasoduodenal; NJ: nasojejunal; PRISM: pediatric risk of mortality.

The gastric volume in mL/kg as well as those in three volume ranges (≤ 0.4 mL/kg, > 0.4 to < 2 mL/kg, and ≥ 2 mL/kg) are listed in Table 2. The median gastric volume and the distribution across the three volume ranges differed significantly between NG- and NJ-fed patients, being greater with NG feeds (P < 0.001). While 81% of NJ patients had gastric volumes ≤ 0.4 mL/kg and none had volumes ≥ 2 mL/kg, more than half of NG patients (53%) had gastric volumes ≥ 2 mL/kg. Only three of 18 patients (18.8%) receiving NJ feeds had a gastric volume greater than 0.4 mL/kg. Additionally, when grading the aspiration risk using the strategy described by Spencer et al [6], there was a higher aspiration risk with NG feeds compared to NJ feeds (33/39 versus 0/18, P < 0.001) (Table 3).

**Table 2:** Gastric Volume Stratified Comparing Nasogastric Versus Post-Pyloric Feedings

	Nasogastric (NG)	Post-pyloric (ND or NJ)
Gastric volume (mL/kg)	2.1 (1.1, 4.5) to 3.7 (4.1)	0 (0, 0.1) to 0.2 (0.4)*
≤ 0.4 mL/kg	3 (9.4)	13 (81.2)*
> 0.4 to < 2 mL/kg	12 (37.5)	3 (18.8)
≥ 2 mL/kg	17 (53.1)	0

The gastric volumes are listed in mL/kg as the median (IQR) and mean (SD) while the distribution based on ≤ 0.4, 0.4–2, and ≥ 2 mL/kg is listed as the number (percentage). The total number of recorded gastric volumes exceeds the number of patients because some patients underwent more than one scan. The volume noted was not listed in seven scans in the NG group and two scans in the NJ group. *P < 0.001. ND: nasoduodenal; NJ: naso-jejunal.

**Table 3:** Risk of Aspiration Stratified by Location of Feeding

Risk of aspiration*	Nasogastric (NG)	Post-pyloric (ND or NJ)
Low risk	6 (15.4)	18 (100)
High risk	33 (84.6)	0

Data are presented as frequency (percentage). The total number of aspiration risk events may exceed the number of patients because some patients underwent multiple scans. Risk of aspiration according to Spencer et al [6] based on characteristic and volume of the gastric contents. *P < 0.001 when comparing risk of aspiration (NG versus post-pyloric). There were 21 encounters with solid or thick fluid on ultrasound. All were NG and all were classified as high risk for aspiration. Additionally, the gastric volumes were high among these 21 as 10 had gastric volumes ≥ 2 mL/kg compared to none in the post-pyloric group. ND: nasoduodenal; NJ: nasojejunal.

## Discussion

Pulmonary aspiration of gastric content is a rare but potentially serious complication during anesthesia and perioperative care, with a reported incidence in the perioperative period ranging from 0.02% to 0.1% [11–14]. Although the incidence of aspiration of gastric contents remains low during the perioperative setting, its consequences can be severe, resulting in the need for unplanned hospital admission, prolonged hospital stay, the need for postoperative ICU admission, postoperative respiratory support including mechanical ventilation, and even death. In order to prevent pulmonary aspiration, practitioners follow standard NPO guidelines that allow sufficient time for gastric emptying [15, 16]. The risk of aspiration and the impact on physiologic function is determined by gastric volume, the pH, and the nature of the gastric contents (liquid, particulate, etc.). Classical teaching states that the severity of aspiration is most severe with the aspiration of gastric volumes greater than 0.2–0.4 mL/kg and/or a pH less than 2.5, while the study of Spencer et al used additional criteria based not only on volume, but also the appearance of the feeds (liquid versus thickened) [6, 17, 18].

Although most studies have focused on perioperative aspiration, risk factors, and means to mitigate these risks, the aspiration of gastric contents is also a concern in critically ill pediatric patients during ICU care. In the ICU setting, other factors may impact gastric volumes including lack of NPO status, feeding routes, and underlying patient factors such as systemic illnesses, gastrointestinal motility, and altered neurologic function. Current clinical practice in adult and pediatric populations in the ICU suggests that post-pyloric (NJ) feeding may reduce the risk of aspiration by bypassing the stomach and promoting continuous small bowel delivery of nutrition. Perioperative data have demonstrated the safety and efficacy of continuing ND feedings during surgery in specific pediatric populations (burn patients) [19]. However, objective data evaluating gastric volume during NJ feeding, especially in mechanically ventilated pediatric ICU patients, have been limited.

A scoping review of 70 studies involving gastric POCUS in acute and critically ill children was published by Valla et al in 2022 [20]. The majority of the studies (67%) used gastric POCUS to assess gastric volume and/or contents. None of these studies compared volumes based on the location of enteral feedings (NG versus post-pyloric). Other studies from the review focused on ultrasound assessments for identification of foreign body ingestion, NG tube placement, diagnosis of hypertrophic pyloric stenosis, and gastric insufflation during mechanical ventilatory support. The authors further noted that none of the studies were conducted in critically ill pediatric patients apart from preterm infants in the neonatal intensive care.

A subsequent study performed in the ICU setting in children compared gastric volumes measured by POCUS and correlated those with gastric residual volumes (GRV) noted by aspiration using an NG tube [21]. GRV measured by aspiration did not correlate with volumes noted by POCUS and failed to empty the stomach in 72% of the 64 children. There was no association noted between GRV and signs of feeding intolerance or gastric POCUS-derived volume. The study may remain relevant as it explores POCUS use in the pediatric ICU clinical setting and supports the feasibility, safety, and potential utility of gastric ultrasound in children while demonstrating the need for ICU-specific investigations.

We could find no other studies comparing different feeding routes (gastric vs. post-pyloric) using ultrasound-derived gastric volumes and aspiration risk assessments. Our study bridges this gap in the literature as the first study using gastric POCUS to compare differences in gastric volume and contents in patients receiving either NG or post-pyloric (ND or NJ) feedings. Gastric volumes were significantly greater in the NG group than in the NJ group. More than half of NG-fed patients (53%) had gastric volumes ≥ 2 mL/kg, whereas none of the NJ-fed patients exceeded this threshold. The majority of the NJ-fed patients (81%) had gastric volumes ≤ 0.4 mL/kg, and none demonstrated sonographic findings consistent with high aspiration risk, when using the grading system described by Spencer et al [6]. A high aspiration risk (grade 2 or solid content present) was identified in 33 of 39 NG-fed patients, but in none of the NJ-fed patients. These results support the premise that post-pyloric feeding minimizes gastric volume and may reduce aspiration risk in critically ill pediatric patients.

We acknowledge specific limitations of the current study. First, the study cohort was relatively small (n = 45), limiting statistical power and generalizability. Because there are limited data quantifying expected gastric volumes in NJ-fed pediatric ICU patients, an evidence-based sample size calculation was not feasible. Second, although baseline demographic (age, weight, and gender) as well as PRISM characteristics were not statistically significant between the two groups, one may consider the differences in demographics and PRISM scores to be clinically significant. The impact of these differences on gastric emptying and the results of the study is unknown. The study protocol included patients who were receiving ≥ 50% goal feeds. Including only patients at full feeds may also impact the results. However, given recruitment issues and non-study related problems that impact escalation of enteral feeds, we did not believe that enrollment would proceed quickly enough if we included only patients at goal feeds. Likewise, we included a small number of patients that were not receiving ≥ 50% of their enteral feeds. Finally, the study population included a limited age range, and the results should not be extrapolated to older adolescents or adult ICU populations without further study.

In summary, the data from our cohort of 45 patients suggest that gastric volumes and the graded aspiration risk are significantly lower in mechanically ventilated pediatric patients receiving post-pyloric feeds compared with NG feeds. These findings propose that withholding NJ feeds for tracheal extubation, operative procedures, or radiologic imaging may be unnecessary because the associated aspiration risk appears minimal and therefore post-pyloric feeds can be continued without interruption during these procedures. Limiting NPO time may serve to improve nutritional intake in critically ill patients. As a small number of our patients did have some residual gastric volumes, it may indicate that a more widespread use of gastric POCUS is warranted in the ICU setting to allow for individualized assessment and risk scoring of patients. Bedside gastric ultrasound offers a simple, noninvasive assessment tool that can assist pediatric intensive care physicians, emergency medicine physicians, and anesthesiologists in individualized decision-making regarding patient safety. In our study cohort, we were able to quickly complete these scans at the bedside in critically ill patients. It was never necessary to stop a scan due to patient-related physiologic issues such as hemodynamic or respiratory instability. This study supports broader use of gastric POCUS during preprocedural and preoperative assessment in the ICU. Future studies with larger cohorts are warranted to confirm these findings and to further define safe feeding practices in this vulnerable population.

## Data Availability

The data supporting the findings of the study are available from the authors.
